# Effects of Metformin Combined With Antifolates on HepG2 Cell Metabolism and Cellular Proliferation

**DOI:** 10.3389/fonc.2022.828988

**Published:** 2022-02-02

**Authors:** Sherouk M. Tawfik, Maha R. A. Abdollah, Mohey M. Elmazar, Hassan A. N. El-Fawal, Anwar Abdelnaser

**Affiliations:** ^1^Department of Chemistry, School of Sciences and Engineering, The American University in Cairo, Cairo, Egypt; ^2^Department of Pharmacology and Biochemistry, Faculty of Pharmacy, The British University in Egypt (BUE), Cairo, Egypt; ^3^The Center for Drug Research and Development (CDRD), Faculty of Pharmacy, The British University in Egypt (BUE), Cairo, Egypt; ^4^Institute of Global Public Health, School of Sciences and Engineering, The American University in Cairo, Cairo, Egypt

**Keywords:** hepatocellular carcinoma, metformin, trimethoprim, methotrexate, antifolates, glycolysis, oxidative phosphorylation, seahorse

## Abstract

Hepatocellular carcinoma (HCC), one of the most prevalent types of cancers worldwide, continues to maintain high levels of resistance to standard therapy. As clinical data revealed poor response rates, the need for developing new methods has increased to improve the overall wellbeing of patients with HCC. Furthermore, a growing body of evidence shows that cancer metabolic changes are a key feature of many types of human malignancies. Metabolic reprogramming refers to cancer cells’ ability to change their metabolism in order to meet the increased energy demand caused by continuous growth, rapid proliferation, and other neoplastic cell characteristics. For these reasons, metabolic pathways may become new therapeutic and chemopreventive targets. The aim of this study was to investigate the metabolic alterations associated with metformin (MET), an anti-diabetic agent when combined with two antifolate drugs: trimethoprim (TMP) or methotrexate (MTX), and how metabolic changes within the cancer cell may be used to increase cellular death. In this study, single drugs and combinations were investigated using *in vitro* assays including cytotoxicity assay (MTT), RT-qPCR, annexin V/PI apoptosis assay, scratch wound assay and Seahorse XF analysis, on a human HCC cell line, HepG2. The cytotoxicity assay showed that the IC_50_ of MET as single therapy was 44.08 mM that was reduced to 22.73 mM and 29.29 mM when combined with TMP and MTX, respectively. The co-treatment of both drugs increased p53 and Bax apoptotic markers, while decreased the anti-apoptotic marker; Bcl-2. Both combinations increased the percentage of apoptotic cells and halted cancer cell migration when compared to MET alone. Furthermore, both combinations decreased the MET-induced increase in glycolysis, while also inducing mitochondrial damage, altering cancer cell bioenergetics. These findings provide an exciting insight into the anti-proliferative and apoptotic effects of MET and anti-folates on HepG2 cells, and how in combination, may potentially combat the aggressiveness of HCC.

## Introduction

Hepatocellular carcinoma (HCC), a liver disease predominant in patients suffering from cirrhosis and chronic liver disease, is a prominent cause of worldwide deaths which occur due to cancer. As the third main cause of cancer world-wide, HCC occurs most frequently in Asia and Africa (1, 2). Due to its high mortality rates, HCC poses as a worldwide health burden. Researchers have informatively suggested that the development of HCC originates from the concept that hepatic stem cells proliferate due to continuous regeneration induced by viral injury (3). Hence, HCC is known for the inflammation, fibrosis, and necrosis of hepatic cells due to the presence of hepatic cirrhosis or hepatitis B virus (HBV), which are vital risk factors in the progression of HCC.

One of the hallmarks of cancer is altered energy metabolism, which is a molecular fingerprint of cancer cells. This metabolic phenotype is defined by an oxygen-independent preferential reliance on glycolysis (the process of converting glucose into pyruvate followed by lactate generation) for energy production. As a result, cancer cells utilize higher levels of glucose to accommodate their altered metabolic state, known as the Warburg effect (4). As cancer cells can become reliant on certain metabolic pathways, new medications targeting these vulnerabilities pose an exciting alternative to cancer therapy.

Metformin (MET) (1,1-dimethyl biguanide), an orally administered drug, is used to decrease the level of blood glucose in patients with non-insulin-dependent diabetes mellitus (NIDDM) by improving insulin sensitivity and decreasing insulin resistance. Recommended as first-line oral therapy in the treatment of diabetes by the American Diabetes Association (ADA), MET exerts its anti-hyperglycemic action by suppressing the production of hepatic glucose, in a process known as hepatic gluconeogenesis (5). As previously stated, MET inhibits complex I of the electron transport chain (ETC) and consequently decreases ATP production by oxidative phosphorylation (OXPHOS). This ultimately disrupts the AMP : ATP ratio, resulting in the activation of 5’ AMP- activated protein kinase (AMPK), an enzyme which constantly detects the cellular energy status by monitoring AMP, ADP, and ATP levels (6). To counteract the improper energy balance upon MET administration, AMPK works to restore ATP levels by impeding biosynthetic pathways and promoting pathways which restore energy balance. AMPK stimulates key processes such as glycolysis, β- oxidation of fatty acids, mitochondrial biogenesis and glucose uptake, while it also switches off protein, glycogen and sterol synthesis in order to salvage ATP (7). AMPK phosphorylates enzymes such as acetyl-CoA carboxylase (ACC) to promote fatty acid oxidation and inhibit fatty acid synthesis, hence altering insulin signaling (8); in addition, AMPK initiates glycolysis through the phosphorylation of phosphofructokinase-2 (PFK-2) (9). Moreover, AMPK promotes the translocation of GLUT4 from intracellular vesicles to the plasma membrane, allowing hepatocytes, skeletal muscles, and adipocytes to take up more glucose (10). The nature of MET in that it allows for the activation of AMPK which consequently affects crucial pathways renders it a potent hypoglycemic drug (11).

Furthermore, one study depicted that in response to MET, *de novo* synthesis of glutathione, a folate-dependent process linked to one-carbon metabolism, was also decreased (12). Accordingly, these findings imply that MET can also act as an antifolate chemotherapeutic drug.

Trimethoprim (TMP) (a synthetic compound), used widely for the treatment of microbial infections, has been shown to inhibit various respiratory and urinary tract pathogens by blocking DHFR (dihydrofolate reductase), an enzyme that catalyzes the reduction of dihydrofolate to tetrahydrofolate (13, 14). Moreover, methotrexate (MTX), which also potently inhibits the synthesis of tetrahydrofolate, the active form of folic acid, was used to treat childhood acute leukemia (15). Interestingly, TMP was shown to cause significant cytotoxicity in bladder cancer cells, suggesting the use of antifolate agents in preventing cancer cell seeding, and hence recurrence (16).

To the best of our knowledge, MET has never been used in conjunction with anti-folates in the treatment of HCC and the impact of this combination on cellular energetics has not been examined using Seahorse analysis. For this reason, in this study, we tested whether MET, when combined with either TMP or MTX, could contribute to abrogating HCC cell survival by combating the compensatory increase in glycolysis due to MET.

## Materials and Methods

### Materials

MET and TMP were kind donations from Nile Company for Pharmaceuticals and Chemical Industries (Cairo, Egypt). MTX vials 50 mg/2 mL (Mylan-Merck Generiques) were purchased, in their formulated commercial preparations, from a community pharmacy (Cairo, Egypt). RevertAid cDNA kit (K1621), PowerUP SYBR Green Master Mix (A25741), mRNA primers (10629186; designed by NCBI primer blast tool), Dulbecco’s Modified Eagle Medium Gibco™ DMEM, High Glucose (41965-039), Fetal Bovine Serum Gibco™ FBS (10270-106), Dimethyl sulfoxide DMSO (67-68-5), Chloroform (HPLC grade; C607SK-1), Isopropanol (HPLC grade; BP26324), and Ethanol (HPLC grade; 64-17-5) were all purchased from ThermoFisher Scientific (MA, USA). QIAzol lysis buffer (79306), RNAse/DNAse free water (129114) were purchased from Qiagen (Hilden, Germany). Penicillin-Streptomycin Mixture Pen/Strep (09-757F), and Phosphate Buffered Saline (1X) (PBS) (17-516Q) were obtained from Lonza-Bioscience (Billerica, MA, USA). Seahorse cell mitochondrial stress test (MST) containing oligomycin, carbonyl cyanide p-trifluoromethoxyphenylhydrazone (FCCP), rotenone + antimycin A (Rot/AA) and glycolytic rate assay kit including Rot/AA and 2-deoxyglucose (2-DG) were obtained from Seahorse Bioscience Inc. (Basel, Switzerland). XF96 cell culture plates, sensor cartridges and XF base medium were also procured from Seahorse Bioscience Inc. Annexin V and propidium iodide were purchased from ThermoFisher Scientific (MA, USA).

### Cell Culture

HepG2 cells (ATCC^®^ HB-8065) were obtained from the National Research Centre (NRC) Cairo, Egypt. HepG2 cells were grown in 75 cm^2^ flasks in a 5% CO_2_ incubator at 37°C, until they reached 80% confluency. HepG2 cells were cultured in Dulbecco’s modified Eagle’s medium (DMEM) high glucose media (Gibco^®^, Thermo Fisher Scientific) supplemented with 10% fetal bovine serum (FBS) (Gibco), 1% Pen-Strep (100 units/mL penicillin, and 100 μg/mL streptomycin (Gibco, MA, USA).

### Cell Viability Assay (MTT Assay)

HepG2 cells were seeded in 96-well plates at a density of 15,000 cells/well. Twenty-four hours later, adherent cells were treated with increasing concentrations of single drugs: MET (12.5, 25, 50, 100, 200 mM), TMP (32.29, 64.58, 129.17, 258.34, 516.67 μM) and MTX (1.56, 3.125, 6.25, 12.5, 25, 50 mM) in fresh DMEM media. The culture medium for dual drugs was composed of increasing concentrations of MET (12.5, 25, 50, 100, 200 mM) and either 516.67 μM TMP or 1.5 mM MTX. Following 24 h incubation with the drugs, culture medium was replaced with 100 μL/well of 10 mg/ml MTT (3-(4,5-Dimethylthiazol-2-yl)-2,5-diphenyltetrazolium bromide) solution prepared in complete DMEM medium. Cells were then incubated for 1 h inside the incubator. MTT media was then removed from the wells and formazan crystals were dissolved in 100 μL/well DMSO. Optical density (absorbance) was measured at 570 nm by using Nano SPECTROstar microplate reader (BMG LABTECH, Ortenberg, Germany). Furthermore, the IC_50_ of the drugs when used as monotherapies or in combination were determined *via* GraphPad Prism software using the non-liner regression analysis.

The isobologram equation was used to determine the combination index (CI) of the tested compounds to elucidate whether the combination was synergistic, additive or antagonistic.


$$Combination\;index\;(CI)=\frac{d1}{D1}+\frac{d2}{D2}$$

where d1 and d2 are the respective MET and either TMP or MTX concentrations used in combination to reach a certain level of growth inhibition, and D1 and D2 are their concentrations capable of causing the same magnitude of growth inhibition when employed alone. The effect of combination is said to be synergistic if CI < 0.8; antagonistic if CI > 1.2; additive if CI ranges from 0.8-1.2 (17).

### RTqPCR

HepG2 cells were seeded in 6-well plates overnight at a seeding density of 250,000 cells/well. Cells were then treated with MET, TMP or MTX and the combinations at concentrations of 20 mM, 516.67 μM and 10 mM, respectively for 48 h. Concentrations of MET and MTX correspond to their respective IC_40_ concentrations, while TMP was used at the maximum concentration possible, given its solubility in DMSO. Total RNA was then isolated using QIAzol Lysis Reagent, Qiagen, Hilden, Germany) according to the manufacturer’s instructions. RNA samples were then assessed to detect purity by measuring the absorbance of the RNA samples at 260 nm (ng/μL) and calculating the A260/280 ratio which was measured using NanoDrop Spectrophotometer (BMG LABTECH, Ortenberg, Germany). cDNA was synthesized using the Revertaid cDNA synthesis kit (K1621; ThermoFisher Scientific, MA, USA), according to the manufacturer’s instructions. Primers sequences, shown in **Table 1**, were generated using the online NCBI primer blast tool and purchased from ThermoFischer (MA, USA). Gene expression levels were calculated as follows: 2^-ΔΔCT^ ± standard error of mean (SEM).

**Table 1:** List of primer sequences and their National Center for Biotechnology Information (NCBI) accession numbers.

Gene name	Primer sequences (5’-3’)	Accession number	Tm (°C)
Bax	F: AAGCTGAGCGAGTGTCTCAAG	NM_138764.5	60.34
	R: CAAAGTAGAAAAGGGCGACAAC		58.11
Bcl-2	F: CTTTGAGTTCGGTGGGGTCA	NM_000633.3	59.89
	R: GGGCCGTACAGTTCCACAAA		60.54
p53	F: CCCTTCCCAGAAAACCTACC	NM_001126118.2	57.49
	R: CTCCGTCATGTGCTGTGACT		60.04
AMPK	F: AAGAAAGTCGGCGTCTGTTC	NM_206907.4	58.50
	R: TTCTGGTGCAGCATAGTTGG		58.17
β-actin	F: AGCACAGAGCCTCGCCTTT	NM_001101.5	61.89
	R: CACGATGGAGGGGAAGAC		56.74

### Cell Apoptosis Assay

The percentage of apoptotic cells was evaluated by using Annexin V and propidium iodide (PI) staining. Cells were grown in T25 flasks and subsequently treated with MET, TMP or MTX and the combinations at concentrations of 20 mM, 516.67 μM and 10 mM, respectively for 48 h. Cells were then harvested, washed with cold 1x PBS, centrifuged three times at 280 x g for 7 min and resuspended in PBS. Aliquots of 100 µL were stained with 5 µL Annexin V‐FITC and 1 µL PI stock (100 µg/mL) and incubated for 15 min at room temperature in the dark. 1x Annexin binding buffer (400 μL) was then added to each sample and analyzed by CytoFlex flow cytometer (Beckman Coulter, CA, USA). according to the manufacturers’ instructions. A minimum of 30,000 events were recorded for each sample. Data analysis was performed in CytExpert software.

### Scratch Wound Assay

Briefly, 10^6^ HepG2 cells were seeded in 6-well plates and allowed to attach overnight. Once the cells reached confluency, a wound was made by scratching the surface with a 200 μL pipette tip held vertically. To remove floating cells, the cells were washed twice with PBS. The cells were then treated with complete DMEM medium and either 3 mM MET, 344.45 μM TMP or 0.2 mM MTX or the combinations MET + TMP and MET + MTX (lower concentrations were used to avoid the detachment of cells). The initial wound area was measured at time 0 using an inverted microscope (magnification power of 400x) (Labomed Inc., LA, CA, USA) connected to a digital camera. The wound distance was then assessed by ImageJ software.

### Seahorse Analysis

Cells were seeded in XF96-well plates (15,000 cells/80 μL medium/well) and left in the incubator to adhere overnight. The next day, the cells were treated with different concentrations MET (3 mM), TMP (86.11 μM), MTX (1.5 mM) and combinations for the glycolytic rate assay (concentrations were lowered to reach optimal basal OCR values). Similar concentrations, as well as higher concentrations were used for the ATP rate assay; MET (6.5 mM), TMP (189.45 μM), MTX (3 mM) and combinations. The higher concentrations only were used for the MST. Twenty-four hours before the start of the experiment, cartridges were soaked in calibrant solution and left in a non-CO_2_ incubator overnight. Before analysis, the culture medium on the plates was removed and cells were washed with 150 μL of XF Seahorse media (supplemented with 2 mM glutamine, 1 mM pyruvate and 10 mM glucose). Then, 180 μL of Seahorse media was added to each of the wells and the plates were incubated for 45 min in a non-CO2 incubator. For each of the assays, compounds were prepared, diluted using XF base medium into designed concentrations and added in the corresponding cartridge ports; (Glycolytic Rate Assay; Rot/AA: 5 μM, 2-DG: 500 mM; ATP Rate Assay; 15 μM Oligomycin and 5 μM Rot/AA; MST; Oligomycin: 15 μM, FCCP: 10 μM and Rot/AA: 5 μM). After calibration, all assays were conducted as per manufacturer’s instructions.

### Statistical Analysis

Each experiment was repeated at least 3 times with 3 to 6 replicates per treatment (representative data are shown in the *Results* section). Data are depicted as means ± SEM for each experiment. Comparisons between treated versus untreated cells for MTT, RT qPCR, apoptosis assay, wound healing assay and Seahorse analysis were done by performing one way ANOVA followed by Tukey *post-hoc* test to assess the statistical significance between multiple groups. A *p*-value < 0.05 was considered statistically significant. SigmaPlot was used to compare the results obtained from the tested compound groups and their relative controls (Version 12.0; Systat Software, Chicago, IL, USA). Graphs were drawn using SigmaPlot software. XF Glycolytic Rate Assay, XF ATP Rate Assay and XF MST parameters were automatically generated using Wave software (Agilent Technologies) to determine OCR (oxygen consumption rate) and ECAR (extracellular acidification rate) values, depicting respiration and acidification rates. Graphs pertaining to the Seahorse data were exported to GraphPad Prism 6 software.

## Results

### Effect of MET, TMP and MTX on HepG2 Cell Viability

**Figure 1:**
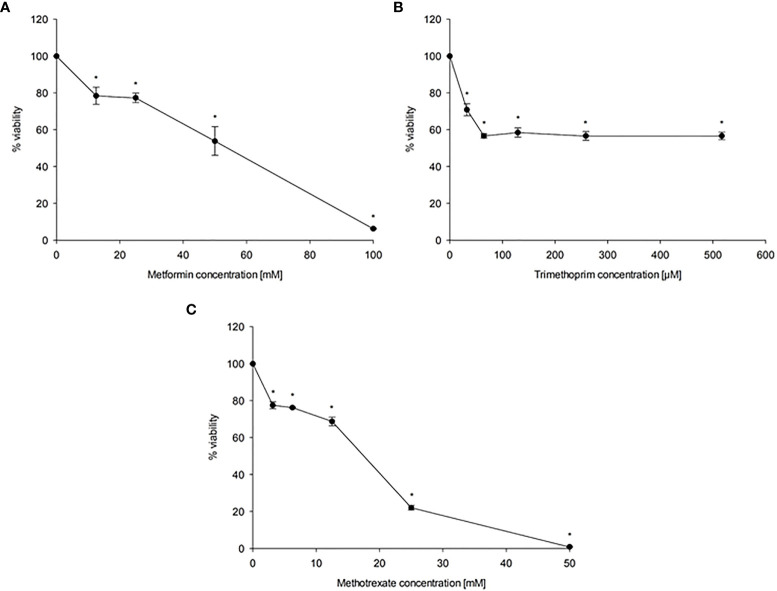
Effect of MET **(A)**, TMP **(B)** and MTX **(C)** on HepG2 cell viability. HepG2 cells were exposed to increasing concentrations of MET (12.5-100 mM), MTX (3.125-50 mM) and TMP (32.29-516.67 μM) for 24 h. The MTT assay was done to assess the inhibitory effects of the tested compounds at the used concentrations. Data are depicted as a percentage of the untreated control. The error bars represent the standard error of mean (SEM) (n=6). Comparisons were made using ANOVA followed by Tukey *post-hoc* test. *, indicates statistical significance when compared to the control.

To evaluate the effect of MET, TMP and MTX on HepG2 cell viability, cells were exposed to increasing concentrations of MET (12.5-100 mM), TMP (32.29-516.67 μM) and MTX (3.125-50 mM). Both MET and MTX significantly reduced HepG2 cell viability in a dose-dependent manner (**Figures 1A, C**). MET individually inhibited cell viability with an IC_50_ value of 44.08 mM, while MTX inhibited cell viability with an IC_50_ value of 14.3 mM. TMP reduced HepG2 cell viability at 516.67 μM, then plateaued at the subsequent concentrations (**Figure 1B**). Notably, due to the limited solubility of TMP in DMSO at non-toxic concentrations, the IC_50_ concentration of TMP was not calculated and is well above the concentrations used in the present study. Hence, all subsequent experiments were conducted using 516.67 μM TMP.

### Effect of MET When Combined With TMP or MTX on HepG2 Cell Viability

**Figure 2:**
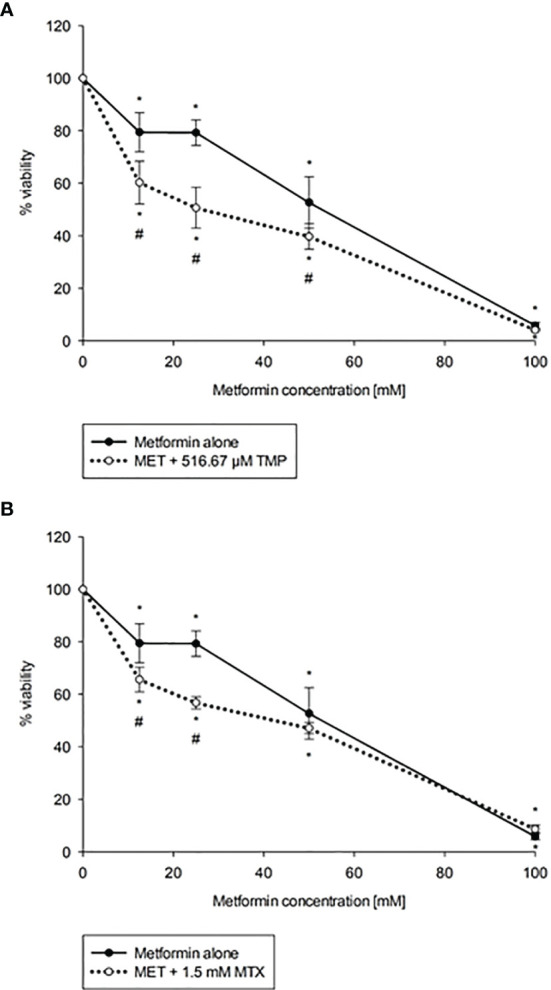
Effect of MET when combined with TMP **(A)** or MTX **(B)** on HepG2 cell viability. Cytotoxicity of various concentrations of MET individually or in combination with TMP or MTX in HepG2 cells were shown above. TMP and MTX increase the cytotoxic effect of MET on HepG2 cells *in vitro*. The MTT assay was done to assess the combinatory effects of MET (12.5-100 mM) and TMP (516.67 μM) or MTX (1.5 mM). Cells were treated with the above concentrations for 24 hours. The IC_50_ of MET was calculated as 44.06 mM, while upon the addition of TMP, the IC_50_ was markedly reduced to 22.73 mM and upon the addition of MTX, the IC_50_ was markedly reduced to 29.29 mM; the error bars represent the SEM (n=6). Comparisons were made using ANOVA followed by Tukey *post-hoc* test. *, indicates statistical significance when compared to the control.

To evaluate the cytotoxicity of MET in combination with both antifolate agents, TMP and MTX, cells were co-exposed with increasing concentration of MET (12.5-100 mM) and either 516.67 μM TMP or 1.5 mM MTX for 24 h. The IC_50_ value of MET was decreased from 44.06 mM to 22.73 mM upon the addition of TMP (CI = 0.998, i.e., an additive effect). As presented in **Figure 2**, all combinations of MET with TMP had more cytotoxic effects compared to MET individually. Furthermore, IC_50_ value of MET was decreased from 44.06 mM to 29.29 mM upon the addition of MTX (CI = 0.763, i.e., a synergistic effect).

### Effect of MET, TMP, MTX and Combinations on Bax, Bcl-2 and p53 mRNA Expression in HepG2 Cells

**Figure 3:**
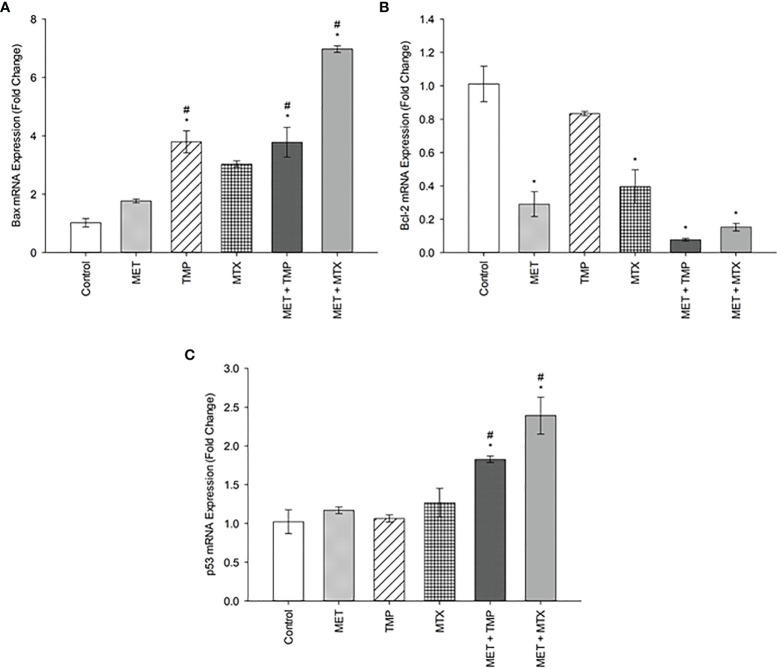
Effect of MET, TMP, MTX and combinations on Bax **(A)**, Bcl-2 **(B)** and p53 **(C)** mRNA expression in HepG2 cells. HepG2 cells were treated for 48 h with MET (20 mM), TMP (516.67 μM), MTX (10 mM), MET + TMP (20 mM + 516.67 μM) or MET + MTX (20 mM + 10 mM). Bax, Bcl-2 and p53 mRNA levels were quantified using qRT-PCR and normalized to β-actin. Data are expressed as mean ± SEM (n=3). Comparisons were made with ANOVA followed by Tukey *post-hoc* test; *; indicates a statistically significant difference between the control and drug-treated groups at P <0.05 versus the control group; #; indicates a statistically significant difference between the MET treated group and other drug-treated groups at *p <*0.05.

The expression levels of apoptosis associated genes, Bax, Bcl-2, p53 were evaluated using the real time quantitative polymerase chain reaction technique. The expression of Bax and p53 were significantly (P<0.05) increased in both combinations, when compared with both the control and cells treated with MET alone, as shown below (**Figure 3**). Contrastingly, the anti-apoptotic gene Bcl-2 decreased significantly, when compared to the control. Our data revealed that Bax was upregulated by 1.77, 3.79, 3.03, 3.78 and 6.20 folds after treatment with MET, TMP, MTX, MET + TMP and MET + MTX, respectively compared to the control. The gene expression of p53 exhibited comparable results and was also upregulated by 1.17, 1.06, 1.27, 1.83- and 2.39-folds following treatment with MET, TMP, MTX, MET + TMP and MET + MTX, respectively compared to the control. In contrast, the gene expression of Bcl-2 was shown to decrease by 0.29, 0.59, 0.4, 0.08 and 0.15 folds when treated with MET, TMP, MTX, MET + TMP and MET + MTX, respectively compared to the control. A comprehensive comparison of the fold changes of each of the tested compounds, singly or in combination, revealed an upregulation of key apoptotic markers and downregulation of an anti-apoptotic gene. Combining MET, with either TMP or MTX, exhibited higher fold change values than that of MET only for both apoptotic markers. These data suggest that the tested compounds in combination significantly trigger apoptosis through the mitochondrial apoptotic pathway.

### Effect of MET, TMP, MTX and Combinations on the Percentage of Apoptosis in HepG2 Cells

**Figure 4:**
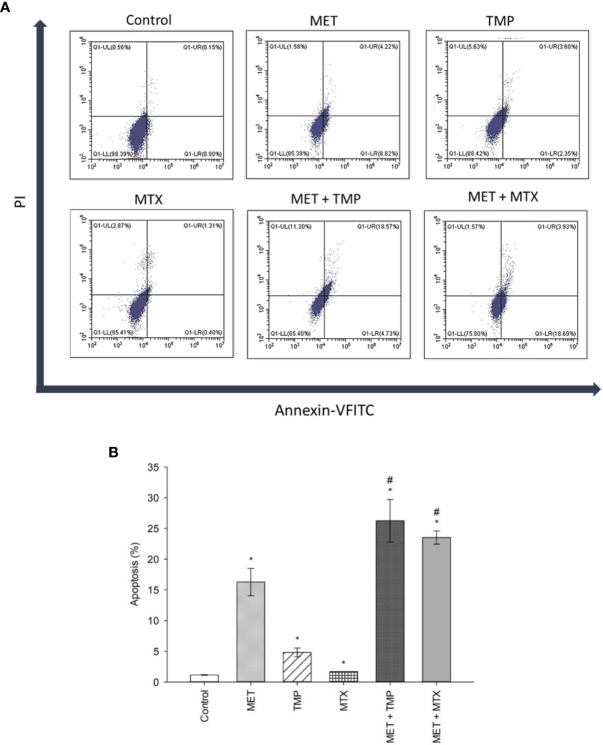
Effect of MET, TMP, MTX and combinations on the percentage of apoptosis in HepG2 cells. **(A)**. Flow cytometry dot plots (Annexin-VFITC against PI) for apoptosis assay. Squares depict populations of cells depending on the presence/absence of phosphatidylserine on the outer surface of the plasma membrane as well as the integrity of the membrane; population of viable cells (LL), early apoptotic cells (LR), late apoptotic cells (UR) and necrotic cells (UL). Annexin V/PI flow cytometry of HepG2 cells treated singly or concurrently with either MET +TMP or MET + MTX for 48 h. Representative data of three independent experiments (*n* = 3) are shown. MET + TMP and MET + MTX combinations significantly induced a higher total percentage of apoptosis in HepG2 cells, compared to single drug treatments. **(B)**. Total percentage of apoptosis (early + late apoptosis) in different treatment groups. Each bar represents the mean of three independent experiments. HepG2 cells were treated for 48 h with MET (20 mM), TMP (516.67 μM), MTX (10 mM), MET + TMP (20 mM + 516.67 μM) or MET + MTX (20 mM + 10 mM). Error bars represent the SEM. Some error bars are too small to be seen. Comparisons were made using ANOVA followed by Tukey *post-hoc* test. *, *p*-value < 0.05 versus control and #, *p*-value < 0.05 versus cells treated with MET only.

To examine the role of apoptosis in the cytotoxic effect of MET, TMP, MTX or combinations, the percentage of apoptotic cells was detected *via* Annexin/PI staining that was measured by the flow cytometry analysis, as previously described. Our results showed that cells co-treated with both combinations induced cell death in HepG2 cells when compared with the control as well as single treatment of MET. MET significantly increased apoptosis at 20 mM and the percentage of viable cells, early apoptotic, late apoptotic and necrotic cells was 85.38 ± 3.88, 8.82 ± 2.78, 4.22 ± 1.08, 1.58 ± 0.08, respectively. TMP at 516.67 μM induced apoptosis and the percentage of viable cells, early apoptotic, late apoptotic and necrotic cells was 88.42 ± 1.37, 2.35 ± 0.45, 3.60 ± 0.81, 5.63 ± 0.16, respectively. It is also of interest that cells treated with MTX (10 mM) did not significantly increase apoptosis. Contrastingly, the percentage of early and late apoptotic cells in the combined treatment of MET and TMP was 4.73 ± 2.15% and 18.57 ± 4.44, respectively. Moreover, the combination of MET with MTX depicted a rise in the percentage of early apoptotic cells, 18.69 ± 1.62, while the percentage of late apoptotic cells was nearly the same, 3.93 ± 0.41. The total percentage of apoptotic cells significantly increased when both drug combinations were used simultaneously (P<0.05), as shown in **Figure 4**, as compared with the control or single treatment. These findings suggest that MET combined with TMP or MTX effectively induced early and late apoptosis in HepG2 cells. Changes in the percentage of total apoptotic cells were consistent with the data obtained from the increase in mRNA expression of key apoptotic markers. Hence, the combination of MET and either TMP or MTX considerably inhibited cell growth in HepG2 cells by inducing apoptosis.

### Effect of MET, TMP, MTX, Alone and in Combination, on HepG2 Cell Migration

Carcinoma cell migration is due to the cancer cells’ ability to undergo various biological processes, specifically related to coordination. As metastasis and angiogenesis are closely related (18), therefore, it was crucial to examine the impact of the drugs on HepG2 cell motility. The ability of MET, TMP, MTX and respective combinations to alter cell migration was analyzed *via* the scratch wound healing assay, which investigates the ability of cells to undergo migration and hence, increase tumorigenesis.

**Figure 5:**
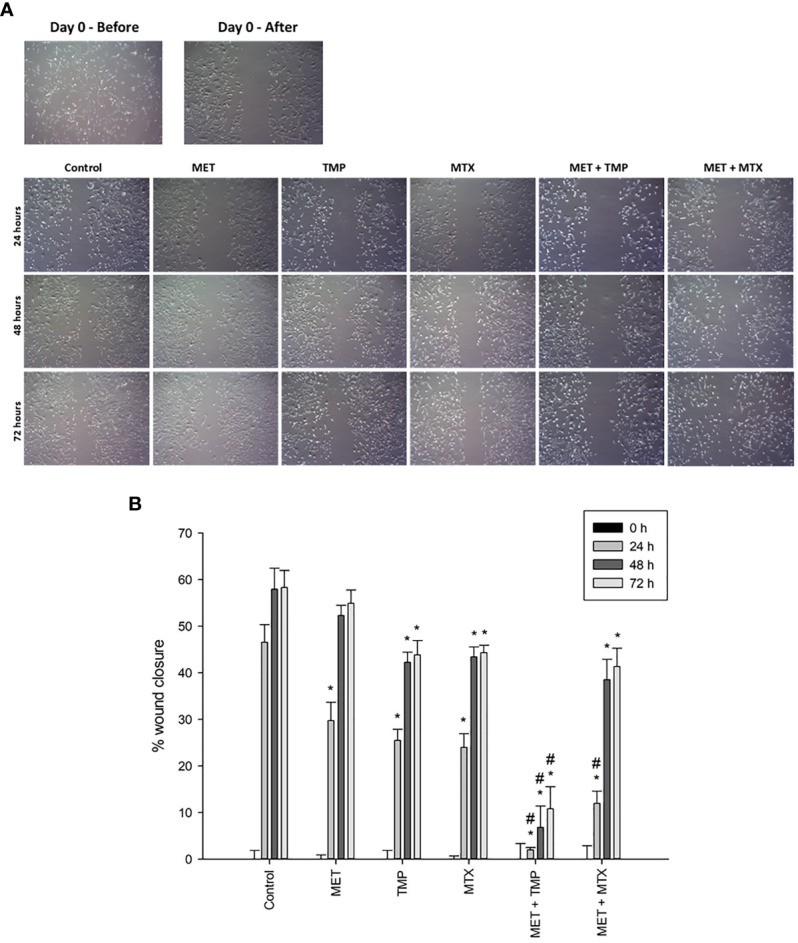
Effect of MET, TMP, MTX, alone and in combination, on HepG2 cell migration. **(A)** Migration of HepG2 cells in response to the treated compounds was determined by the wound healing assay at 24, 48 and 72 h using an inverted microscope at 400x magnification. **(B)** Percentage of wound closure was calculated at 0, 24, 48 and 72 h by measuring the gap width with respect to the initial scratch area. Error bars represent the SEM. Comparisons were made using ANOVA followed by Tukey *post-hoc* test. *, *p*-value < 0.05 versus control at equal time points and #, p-value < 0.05 versus cells treated with MET only at equal time points.

The effects of MET, TMP, MTX and combinations on cell migration were observed in HepG2 cell line. Cells were cultured in 6-well plates and gaps were made using a 200 μL tip to ensure a cell-free gap in each well. HepG2 cells were then treated with 3 mM MET, 344.45 μM TMP or 0.2 mM MTX or the combinations (MET +TMP) and (MET+MTX) and incubated for up to 72 h. Images were taken every 24 h for three consecutive days. Co-presence of MET and TMP resulted in a significantly lower percentage of wound closure when compared to the presence of MET (3 mM) from 29.75 ± 3.94% to 1.97 ± 0.53% at 24 h, from 52.29 ± 2.2% to 6.79 ± 4.56% at 48 h and from 54.93 ± 2.83% to 10.8 ± 4.70% at 72 h, respectively (**Figure 5**). Contrastingly, MET when combined with MTX inhibited cell migration, to a much less extent when compared to MET alone; from 29.75 ± 3.94% to 11.94 ± 2.61% at 24 h, from 52.29 ± 2.2% to 38.5 ± 4.38% at 48 h and from 54.93 ± 2.83% to 41.35 ± 3.92% at 72 h, respectively. TMP and MTX alone significantly decreased HepG2 cellular migration, when compared to the control at the same time points. TMP alone resulted in a percentage of wound closure of 25.45 ± 2.42% at 24 h, 42.24 ± 2.19% at 48 h and 43.86 ± 3.07% at 72 h. Moreover, MTX caused a percentage of wound closure of 23.99 ± 2.92% at 24 h, 43.43 ± 2.11% at 48 h and 44.31 ± 1.63% at 72 h.

### Effect of MET, TMP and MTX, Alone and in Combination, on Rates of Basal and Compensatory Glycolysis in HepG2 Cells

**Figure 6:**
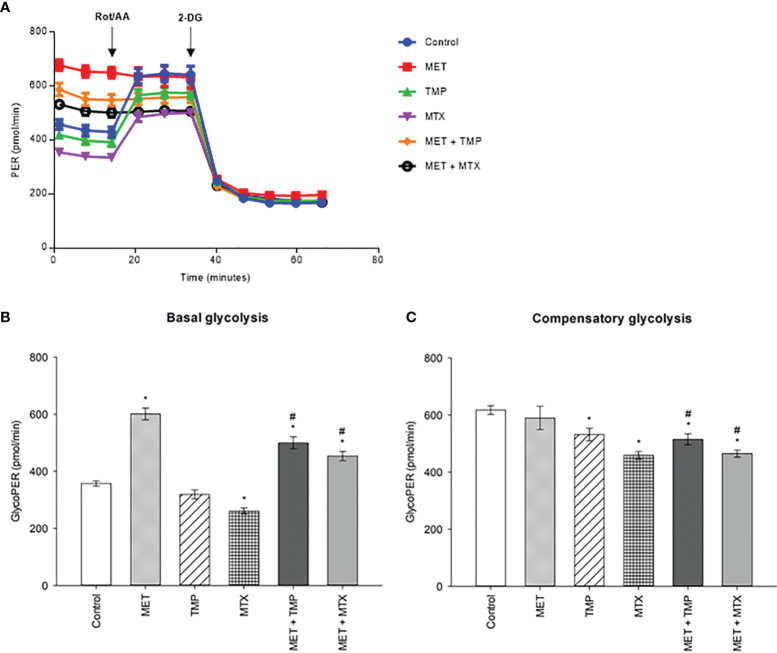
Effect of MET, TMP and MTX, alone and in combination, on rates of basal and compensatory glycolysis in HepG2 cells. Cells were seeded in Seahorse tissue culture microplates, treated with MET (3 mM), TMP (86.11 μM), MTX (1.5 mM), MET + TMP (3 mM + 86.11 μM) or MET + MTX (3 mM + 1.5 mM) for 24 hours and examined by the Glycolytic Rate Assay in which Rot/AA and 2-DG were added as shown above. **(A)** Representative Glycolytic Rate Assay profile. **(B)** Calculated basal glycolytic proton efflux rate (glycoPER). **(C)** Calculated compensatory glycolytic proton efflux rate (glycoPER). Data are expressed as mean ± SEM (n=6). Comparisons were made using ANOVA followed by Tukey *post-hoc* test. *, *p*-value < 0.05 versus control and #, *p*-value < 0.05 versus cells treated with MET only.

To examine if the tested compounds influence the Warburg effect, MET, TMP, MTX, MET + TMP and MET + MTX treated groups were examined in terms of rate of glycolysis. MET alone, or in combination, activated glycolysis up to the maximum level, as shown by the insensitivity to oligomycin. MET caused an increase in basal glycolysis depicted by a 68% increase, when compared to the control (**Figure 6**). Contrastingly, TMP and MTX alone decreased basal glycolysis rates by 11% and 27%, respectively, also when compared to the control. Of significance, co-treatment of MET and TMP or MET and MTX, decreased basal glycolysis rates by 17% and 25%, when compared to MET alone. Furthermore, MET caused a slight decrease in the rates of compensatory glycolysis by 4%, when compared to the control, while TMP and MTX decreased compensatory glycolysis by 14% and 26%, respectively. Rates of compensatory glycolysis were significantly decreased upon co-therapy of MET and TMP or MET and MTX by 13% and 21%, respectively, when compared to MET alone. These data bring to light the suggestion that both TMP and MTX significantly combat the MET-induced shift in glycolysis.

### Effect of MET, TMP and MTX and Combinations on the Total ATP Production Rate in HepG2 Cells

**Figure 7:**
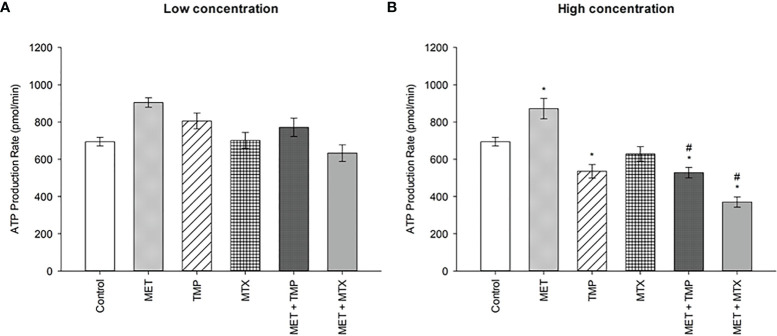
Effect of MET, TMP and MTX and combinations on the total ATP production rate in HepG2 cells. **(A)** HepG2 cells were treated for 24 h with MET (3 mM), TMP (86.11 μM), MTX (1.5 mM), MET + TMP (3 mM + 86.11 μM) or MET + MTX (3 mM + 1.5 mM). **(B)** HepG2 cells were treated for 24 h with MET (6.5 mM), TMP (189.45 μM), MTX (3 mM), MET + TMP (6.5 mM + 189.45 μM) or MET + MTX (6.5 mM + 3 mM) and measured by Seahorse XF Real-Time ATP rate assays. Data are expressed as mean ± SEM (n=6). Comparisons were made using ANOVA followed by Tukey *post-hoc* test. *, *p*-value < 0.05 versus control and #, *p*-value < 0.05 versus cells treated with MET only.

To analyze living cells, sub-IC_50_ values were used to measure the total ATP production rates in HepG2 cells. Two concentrations were used for the tested compounds, alone and in combination (**Figure 7**). At low and high concentrations, MET increased total ATP production rates by 30% and 26%, respectively, when compared to the control. Contrastingly, TMP and MTX (at low concentrations) induced an increase in total ATP production rate by 16% and 1%, respectively, when compared to the control. Contrastingly, TMP induced a decrease in total ATP production by 23% at high concentrations, while MTX, similarly, induced a decrease by 9%, when compared to the control. MET + TMP and MET + MTX significantly decreased ATP production in a dose dependent manner compared with MET alone at both low and high concentrations, respectively; MET + TMP (15% and 39%) and MET + MTX (30% and 58%).

### Effect of MET, TMP and MTX, Alone and in Combination, on the Glycolytic and Mitochondrial ATP Production Rates in HepG2 Cells

**Figure 8:**
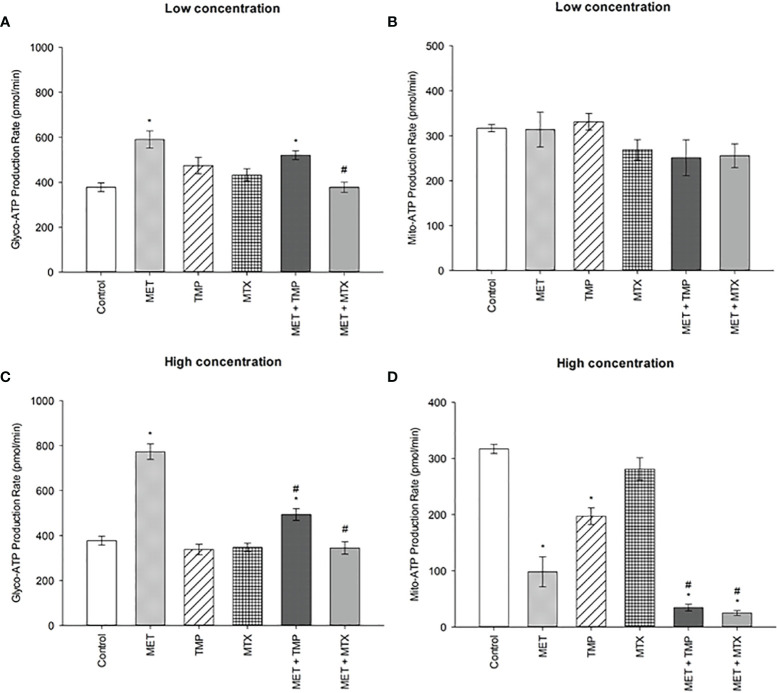
Effect of MET, TMP and MTX, alone and in combination, on the glycolytic and mitochondrial ATP production rates in HepG2 cells. **(A, B)** Glycolytic and mitochondrial ATP production rates decreased upon co-treatment of MET with TMP or MTX. HepG2 cells were treated for 24 h with MET (3 mM), TMP (86.11 μM), MTX (1.5 mM), MET + TMP (3 mM + 86.11 μM) or MET + MTX (3 mM + 1.5 mM). **(C, D)** Percentage of ATP production from glycolysis and mitochondria significantly decreased upon combination of MET with either TMP or MTX, when compared to MET only. HepG2 cells were treated for 24 h with MET (6.5 mM), TMP (189.45 μM), MTX (3 mM), MET + TMP (6.5 mM + 189.45 μM) or MET + MTX (6.5 mM + 3 mM) and measured by Seahorse XF Real-Time ATP rate assays. Data are expressed as mean ± SEM (n=6). Comparisons were made using ANOVA followed by Tukey *post-hoc* test. *, *p*-value < 0.05 versus control and #, *p*-value < 0.05 versus cells treated with MET only.

Consistent with the percentage of basal and compensatory glycolysis rates depicted in **Figure 6**, treatment of MET led to an increase in rate of glycolysis, while the combinations led to a decrease in glycolysis rate (**Figure 8**). Both concentrations of MET increased the glycolytic ATP production rate by 57% and 105%, respectively, when compared to the control. TMP, on the other hand, increased glycolysis by 26% at low concentration and decreased the rate of glycolysis by 11% at higher concentrations. Similarly, MTX increased glycolysis by 15% when administered at a low concentration, while decreased the glycolytic rate by 8% at higher concentrations, when compared to the control. Combining MET and TMP or MET and MTX at low concentrations decreased the rate of glycolysis by 12% and 36%, respectively, when compared to MET alone. Interestingly, both combinations (MET + TMP and MET + MTX) effectively led to a more prominent decrease in the rate of glycolysis at higher concentrations; 36% and 55%, respectively when compared to MET alone. Hence, our results confirmed the findings obtained from the Glycolytic Rate Assay depicted above.

Of significance, the rate of mitochondrial ATP production was also impacted as a result of drug treatments. MET and MTX (at low concentrations) decreased mito-ATP production by 0.92% and 13%, respectively when compared to the control. Contrastingly, TMP slightly increased the mito-ATP production rate by 4%. Both combinations, on the other hand, declined these rates by 21% and 19%, when compared to MET alone. Furthermore, MET, TMP and MTX (at high concentrations) elucidated a higher decrease in mito-ATP production by 69%, 38% and 11%, respectively, when compared to the control. Of note, co-treatment of MET + TMP and MET + MTX further led to a decrease in mitochondrial ATP production by 65% and 75%, respectively, when compared to MET alone.

### Effect of MET, TMP, MTX and Combinations on AMPK mRNA Expression in HepG2 Cells

MET is well known to inhibit complex I of the mitochondrial respiratory chain, resulting in a decrease in the ATP/AMP ratio, and consequent AMPK activation (19, 20). Therefore, we decided to monitor the gene expression of AMPK following drug incubation.

**Figure 9:**
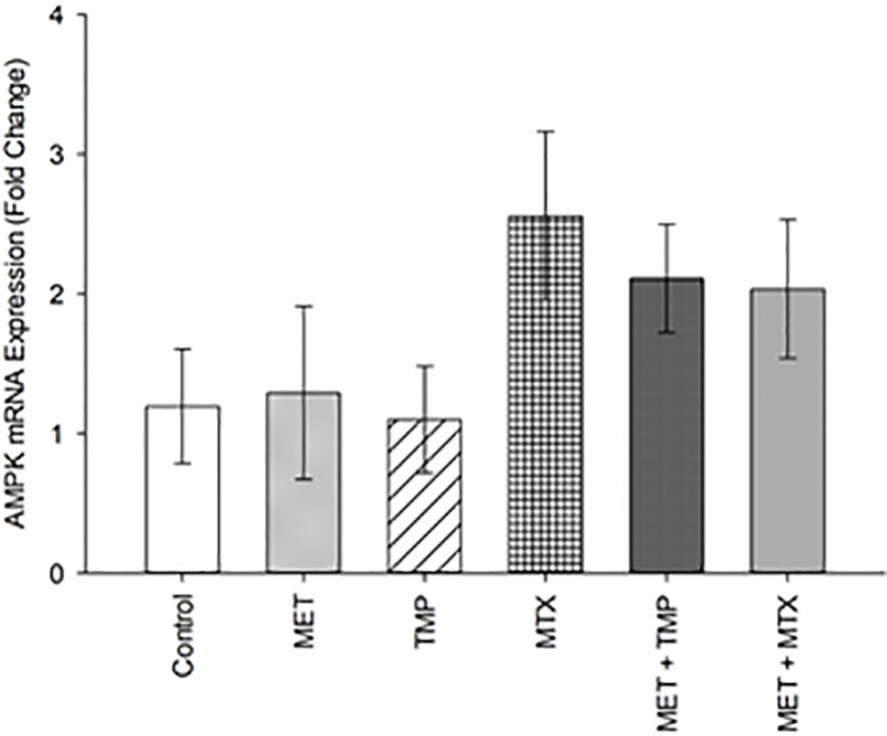
Effect of MET, TMP, MTX and combinations on AMPK mRNA expression in HepG2 cells. HepG2 cells were treated with MET (6.5 mM), TMP (189.45 μM), MTX (3 mM), MET + TMP (6.5 mM + 189.45 μM) or MET + MTX (6.5 mM + 3 mM). AMPK mRNA levels were quantified using qRT-PCR and normalized to β-actin. Data are expressed as mean ± SEM. (n=3).

Our data revealed that AMPK was upregulated by 1.29, 1.10, 2.55, 2.11 and 2.03 folds after treatment with MET, TMP, MTX, MET + TMP and MET + MTX, respectively compared to the control (**Figure 9**). The increasing pattern in terms of fold change confirmed the results obtain *via* the ATP Rate assay, though none of the values above were considered significant.

### Effect of MET, TMP, MTX, Alone and in Combination, on Mitochondrial Bioenergetics

Combining MET to either TMP or MTX leads to inhibition of mitochondrial bioenergetics.

**Figure 10:**
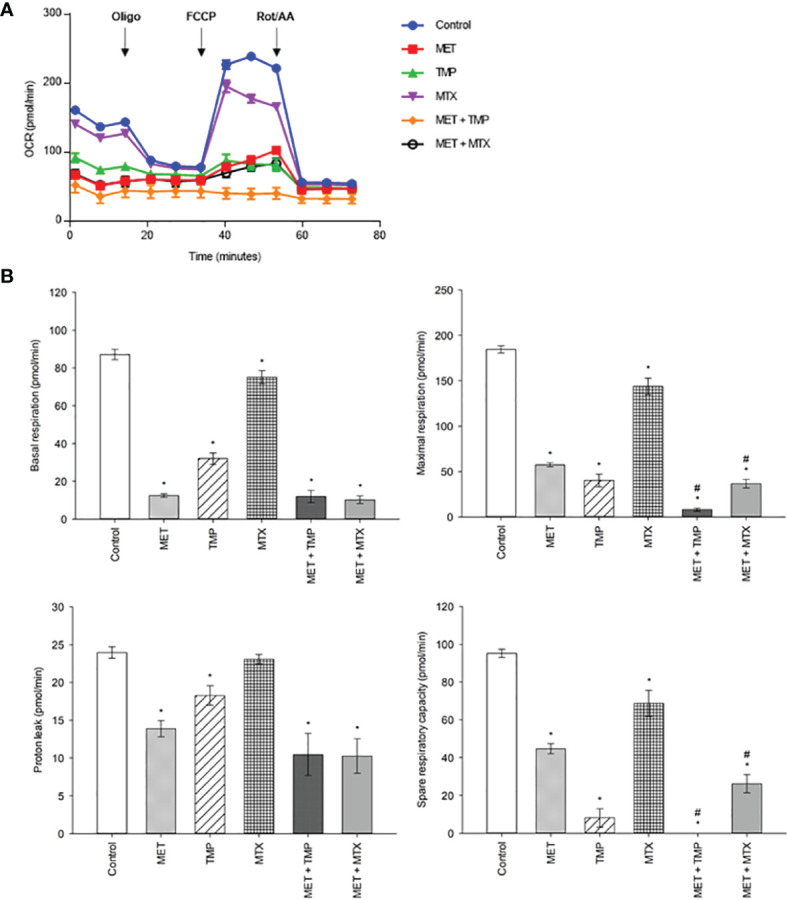
Effect of MET, TMP, MTX, alone and in combination, on mitochondrial bioenergetics. Combining MET to either TMP or MTX leads to inhibition of mitochondrial bioenergetics. **(A)** The effect of treatment of MET, TMP, MTX and combinations on the rate of mitochondrial respiration (OCR) in HepG2 cells after 24 h. TMP and MTX combined with MET induced mitochondrial dysfunction in HepG2 cells. A decrease in OCR of cells is seen following combination therapy, when compared to the control. **(B)** Basal respiratory rate, maximal respiration, proton leak and spare respiratory capacity of HepG2 cells following treatment of MET (6.5 mM), TMP (189.45 μM), MTX (3 mM), MET + TMP (6.5 mM + 189.45 μM) or MET + MTX (6.5 mM + 3 mM) for 24 **(h)** Following measurements of basal respiration, oligomycin (1.5 µM), FCCP (1 mM) and Rot/AA (0.5 µM) were injected to measure key mitochondrial parameters. The combination treatment clearly caused a significant decrease in mitochondrial function in HepG2 cells. Data are expressed as mean ± SEM (n=6). Comparisons were made using ANOVA followed by Tukey *post-hoc* test. *, *p*-value < 0.05 versus control and #, *p*-value < 0.05 versus cells treated with MET only.

As MET has been previously known to inhibit OXPHOS, the tested compounds were examined alone and in combination (at high concentrations) to further investigate the effects of the combinations on mitochondrial function using the MST. HepG2 cells were incubated with MET, TMP, MTX or respective combinations at sub-IC_50_ concentrations for 24 h. Following incubation, cells were incubated in a non-CO_2_ incubator for 45 min and then examined using the Seahorse XFe96 Analyzer. Real-time measurements of OCR were measured (**Figure 10**). MET caused a decrease in mitochondrial function as elucidated by a sharp reduction in mitochondria basal activity (calculated as the difference between basal OCR and non-mitochondrial OCR), maximal respiration (maximal OCR after the addition of the upcoupler FCCP), proton leak (remaining basal respiration not coupled to ATP production) and spare respiratory capacity (the difference between basal and maximal rates) by 86%, 69%, 42% and 53%, respectively, when compared to the control. Similarly, TMP alone reduced basal respiration, maximal respiration, proton leak and spare respiratory capacity by 64%, 78%, 24% and 91%, respectively, when compared to untreated cells. Furthermore, MTX decreased the above assessed parameters, in the same order, by 16%, 22%, 4% and 28%, respectively, when compared to the control. Interestingly, the mitochondrial inhibitory functions of MET were increased upon the addition of either TMP or MTX. Following 24 h incubation with MET + TMP, cells depicted a basal respiration and maximal respiration reduction by 6% and 86%, respectively, when compared to MET alone. MET + TMP also induced a decrease in proton leak, which reached 25%, while the spare respiratory capacity was completely abolished at 24 h. Moreover, MET when combined with MTX also exhibited a decrease in basal and maximal respiration by 18% and 36%, respectively, when compared to MET alone. In congruence with these findings, the proton leak and spare respiratory capacity were also reduced by 26% and 41% upon co-treatment of tested compounds, compared to MET alone. These data suggest that TMP and MTX may potentiate the detrimental action of MET on mitochondrial function in HepG2 cells.

## Discussion

Inhibiting glycolysis appears to be a logical treatment strategy for cancer cells which rely heavily on this pathway. Due to cancer cells’ metabolic adaptability, combining drugs that target different metabolic pathways to acquire better therapeutic activity is necessary. Furthermore, clinical evidence has emerged that the use of a single therapeutic agent for treatment has proven to be less effective in preventing the recurrence of various cancers (21). Moreover, cancer cells are known to exemplify resistance to pharmacological therapeutics through signaling pathways, thereby increasing mortality rates in liver cancer patients (22). Furthermore, HCC, one of the most common types of cancers worldwide, portrays poor prognosis in currently existing treatment options. Combination therapy hence provides an exciting alternative for improving therapeutic outcomes and reducing recurrence in HCC. Additionally, repurposing FDA approved drugs provides a more economical approach to drug development. The co-treatment of drugs that alter cancer cell metabolism and antifolate agents may yield more effective results (23).

MET, used as first-line treatment of type 2 DM, is a safe and economical therapeutic agent which stands to be one of the most widely prescribed drugs worldwide (24, 25). Several studies have shown the potential of MET as a chemotherapeutic agent in various cancer types, such as breast cancer, lung cancer, gastric and colorectal cancer (26–28). Mechanistic investigations on the mode of action of MET have also demonstrated the ability of MET to inhibit cancer cell proliferation and induce apoptosis *in vitro* in a number of human cancer cell lines (29, 30). In another study, MET served to combat thyroid cancer in a dose dependent manner (31). Furthermore, MET significantly inhibited breast and lung cancer cell proliferation when combined with Paclitaxel by inducing AMPK activation and inhibiting mTOR levels (30).

MET has shown to be more effective in combination with other anti-cancer agents when compared to single therapy; i.e. doxorubicin and cisplatin (32). However, to the best of our knowledge, MET has not been previously investigated with either TMP or MTX on HCC. In the present study, the molecular mechanisms associated with the cytotoxic effects of MET + TMP and MET + MTX were tested to assess the effectiveness of the respective combinations in the treatment of HCC.

In the present study, we found that treatment of HepG2 cell line with either MET, TMP or MTX directly inhibits cell survival. In addition, the co-treatment of MET and either TMP or MTX effectively inhibited HepG2 cell survival at sub-IC_50_ concentrations, causing a reduction in the IC_50_ concentration of MET alone. Our findings are consistent with previous studies that depicted the cytotoxic effects of MET and WP 631 (a structural analogue of doxorubicin) on HepG2 cells (33).

Alternatively, the combination of WP 631 and sitagliptin (a dipeptidyl peptidase-4 inhibitor used for the treatment of type 2 diabetes) did not enhance the cytotoxic effects of WP 631 on HepG2 cells. Moreover, our results are in strong agreement with previous reports of MET in combination with potential chemotherapeutic agents on various breast cancer cell lines (34). Another study also reported that the combined treatment of MET with aloin (an extract of Aloe vera) inhibits HCC growth *in vitro* and *in vivo* (35). Their findings were in uniformity with our results in that MET also elucidated a stronger anti-cancer effect when compared to either drug alone; however, upon combination, the added therapeutic agent increased the cytotoxicity of MET in HepG2 cells. Additionally, MET and curcumin were reported to have inhibited the growth, metastasis and angiogenesis of HCC (36). Co-treatment of MET and sorafenib (an FDA approved drug for the treatment of advanced HCC) also effectively decreased the growth of HCC cells, when compared to each drug alone (37–39). MET was also previously reported to have improved the sensitivity of ovarian cancer cells to MTX, compared with the chemotherapeutic agent MTX alone (40). Furthermore, another study reported that MET when used in combination with rapamycin decreased cancer cell viability in HepG2 cells by inducing cell apoptosis (41).

All subsequent experiments were carried out by the calculated sub-IC_50_ values of the tested compounds, alone and in combination. Our findings were consistent with the previously mentioned studies which were conducted on HepG2 cells confirming that the combination of MET with both tested antifolate compounds dramatically inhibited cell viability, when compared with single therapy of MET alone. To examine the effect of the tested chemotherapeutic agents on induction of apoptosis, we investigated the effect of MET, TMP, MTX and respective combinations on the expression of p53, Bax and Bcl-2 on HepG2 cells.

Apoptosis is initiated *via* two signaling pathways; intrinsic or extrinsic (42, 43). Bax, Bcl-2 and p53 are associated with mitochondrial-associated intrinsic apoptosis (44). Bax induces apoptotic cell death by forming pores in the mitochondrial outer membrane. Cytochrome C molecules, which are proapoptotic factors, are then able to translocate from the mitochondria to the cytoplasm, disabling the production of ATP and initiating proteolytic caspase cascade (45). Numerous studies have also suggested that the levels of p53, a tumor suppressor gene, is involved in cell cycle regulation and DNA repair. Once activated, p53 has also been seen to induce AMPK-mediated cell cycle arrest (46). In this aspect, the combined treatment of cells with MET and either TMP or MTX increased p53 and Bax gene levels, while decreasing Bcl-2 levels. Hence, the co-treatment of MET with the antifolate agents (TMP or MTX) on HepG2 cells enhanced the decrease in cancer cell viability through changes in levels of genes involved in the intrinsic pathway of apoptosis; p53, Bax and Bcl-2. When compared to MET alone, both combinations stimulated apoptosis more prominently.

Our results are in agreement with other findings that indicated that an increase in the levels of Bax and a decrease in the Bcl-2 levels are linked to cytochrome C release and increased apoptosis (47). Similar to the findings presented in the section 3, the combination of MET and DSF-Cu (an FDA approved repurposed drug used for the treatment of alcohol abusers) also increased the expression of key apoptotic markers, Bax and p53, but at lower concentrations of MET (48). The decrease in MET concentration may be due to the difference in experimental conditions and diverse cell line used. In another recent study, MET when combined with EGCG (epigallocatechin-3-gallate, a polyphenol present in green tea), increased the levels of caspase-3 and decreased levels of survivin, thereby significantly promoting apoptosis in HCC cells (49), Additionally, another study showed that the co-treatment of HepG2 cells with ATO (arsenic trioxide, a therapeutic agent used in the treatment of acute promyelocytic leukemia) potentiated the anti-HCC efficacy of ATO and increased apoptosis *in vitro* by decreasing the levels of Bcl-2 (50).

Apoptosis was also evaluated in HepG2 cells by flow cytometry after double staining with Annexin V and PI. The percentage of total apoptotic cells (early and late apoptosis) in cells treated with the combined therapy was also consistent with the increase in gene expression of pro-apoptotic molecules. The presence of apoptotic or necrotic cells is not the only indication of cytotoxicity of the tested combinations; for this reason, the impact of treatments on the migration of HepG2 cells was also examined.

In line with the previous results, drug combinations potentially inhibited migration of HepG2 cells *via* decreasing proliferation and increasing the percentage of apoptotic cells. Cell migration, a mechanism involved in the metastatic progression of cancer, is associated with lack of cell-cell adhesion, accelerated migration and cancer cell invasion (51). While higher concentrations of MET elucidate both a decrease in cancer cell viability and induction of apoptosis, the effect of MET on cancer cell migration is prominent even at lower doses (3 mM causing an inhibition in the wound healing assay) (**Figure 5**), suggesting that MET targets various pathways to differing extents.

Our findings suggest the potential effects of MET and combinations on the inhibition of migration of HepG2 cells. Interestingly, upon the addition of MTX to MET, cancer cell migration was not significantly altered, suggesting a potential antagonistic role of MET on the effect of MTX on HepG2 cell migration. Contrastingly, our data suggest a strong effect of MET + TMP on migration by significantly reducing wound closure, demonstrating that the sub-IC_50_ concentrations of both drugs may be significant in preventing the metastasis of HCC.

In one study, MET slightly increased HER+ cell migration, while the combination of MET with aspirin inhibited cancer cell migration in triple-negative breast cancer as well as MCF-7 cell lines, in alignment with our results. To the contrary, the co-treatment of MET with aspirin did not induce a significant change in MDA-MB-231 and SK-BR-3 cell lines (52). Additionally, another study depicted a reduction in MDA-MB-231 cell migration upon treatment of the same concentration of MET used in the present study (53). Therefore, the effects of MET on cancer cell migration, alone or in combination, vary according to the cancer cell type.

Recent studies have shown that metabolic alterations are crucial for the survival and proliferation of cancer cells. There is emerging evidence that glycolysis and OXPHOS are essential drivers in cancer cell metastasis (54). Enzymes involved in glycolysis have been shown to play a key role in tumor migration and invasion. Phosphoglucose isomerase (PGI), for instance, is a cytosolic enzyme that catalyzes the conversion of glucose-6-phosphate to fructose-6-phosphate in the second step of glycolysis (55). Studies have depicted that PGI is an autocrine motility factor (AMF) and a tumor-secreted cytokine, which induces cell migration *in vitro* and metastasis *in vivo* (56). Hence, PGI/AMF is required for tumor cell migration, invasion, and metastasis, and has anti-apoptotic effects on malignant cancer cells, as well as other roles in tumor progression (57, 58). Furthermore, by altering the cancer microenvironment *via* accumulating lactate, excessive glycolysis has been shown to enhance cancer stem cell phenotypic, angiogenesis, migration, and immune evasion (59, 60). Additionally, growth factor-stimulated or cancerous cells require an adequate amount of nutrients to meet the metabolic demands of cellular migration and proliferation. In the absence of nutrition, metabolic checkpoints are triggered, resulting in cell cycle arrest and activation of the intrinsic apoptotic cascade *via* a mechanism involving the Bcl-2 family of proteins (61).

For this reason, the effect of the tested compounds, alone and in combination, on mitochondrial function was assessed. Cancer cells tend to utilize glycolysis to produce ATP, while also maintain OXPHOS for energy production. Since tumors proliferate more quickly than normal tissues, they require a larger amount of ATP as a source of energy. Therefore, drugs targeting the metabolic pathway of cancer cells pose as potential chemotherapeutics. MET has been widely known to inhibit mitochondrial function, by inhibiting complex I of the ETC (62). Consequently, cancer cells treated with MET exhibit an increase in rate of glycolysis as a compensatory mechanism in the aim of increasing ATP production (63). However, if the compensatory increase in glycolysis fails to meet the cellular ATP demands, AMPK is activated to potentiate catabolic metabolism, while inhibiting anabolic processes (64–66). AMPK phosphorylation and activation causes acetyl-CoA carboxylase (ACC), one of the most well-studied AMPK targets, to be phosphorylated and inactivated, resulting in the reduction of lipogenesis (67, 68). Furthermore, MET increases the levels of AMP, leading to the inhibition of adenylate cyclase (69). MET also inhibits mTOR signaling, leading to decreased protein synthesis (70, 71). Overall, MET causes a reduction in cellular energy status, resulting in a decrease in ATP-consuming processes. This may result in a cytostatic condition in proliferating cells, which is associated with lower proliferation and could explain the anti-cancer effects of MET. In a similar vein, cancer cells that are unable to compensate for their reduced energy status may undergo apoptosis, rendering MET cytotoxic (72, 73). Hence, preventing this compensatory metabolic event would directly impact cancer cell survival.

We aimed to test whether MET in combination with antifolates would inhibit the growth of cancer cells, by decreasing the MET-induced increase in glycolysis, hence, potentiating cell death. As the Seahorse XFe96 Analyzer measures glycolytic and mitochondrial parameters in real-time, optimization of the respective drug concentrations used was done to ensure adequate measurements of parameters within the allowed range (20-200 OCR). After 24 h of incubation with the tested compounds, alone or in combination, the glycolytic rate assay was performed to examine the rate of glycolysis in these cells. MET significantly increased the basal rate of glycolysis, when compared to the control. These results are consistent with published literature reporting the MET-associated inhibition of OXPHOS and hence, rise in glycolysis (74). Furthermore, both TMP and MTX alone decreased basal glycolysis rates, when compared to the control. Combined treatment of MET and TMP or MTX exhibited a significant decrease in the basal rate of glycolysis in HepG2 cells, when compared to MET alone (**Figure 6B**).

To further confirm the findings obtained from the glycolytic rate assay, we further went on to perform the ATP rate assay. MET alone induced an increase in the total ATP production rate in HepG2 cells (**Figure 7**). Moreover, both TMP and MTX increased the total ATP production when used alone. Consistent with our previous findings, both combinations decreased the percentage of total ATP production in HepG2 cells and significantly impacted the percentage of ATP production produced *via* glycolysis. The ATP rate assay also shed light on the effect of tested compounds on the mitochondrial ATP production. Both combinations elucidated a decrease in the rate of ATP production *via* the mitochondria (**Figure 8**), yet further analysis was needed to confirm these results. AMPK mRNA expression was also evaluated as AMPK values increase with decrease in ATP levels (75). Both combinations depicted an increase in AMPK levels, confirming our findings, though they were not significant (**Figure 9**). However, evaluation of the different AMPK subunits phospho-isoforms at the protein level would further elucidate the role of AMPK in controlling cancer cell bioenergetics.

We then conducted the MST on HepG2 cells treated with the compounds alone and in combination for 24 h. Following MST, data confirmed that MET induced mitochondrial injury, consistent with previous findings. Interestingly, both TMP and MTX also inhibited mitochondrial function, but to a lesser extent. Co-treatment of MET and either antifolate resulted in the significant decrease of OCR, compared to the control. Basal respiration as well as proton leak decreased, but not significantly, when compared to MET alone. Contrastingly, maximal respiration and the spare respiratory capacity significantly declined, compared with MET treatment alone (**Figure 10B**). In conclusion, these data suggest that MET in combination with antifolates (TMP or MTX) impact the energy production in HepG2 cells *via* two main pathways: OXPHOS and glycolysis. Furthermore, the powerful anti-metastatic characteristics of the tested compounds are likely a result of the ability of both combinations to inhibit the mitochondrial bioenergetics. These combinations might be particularly useful in preventing liver cancer metastases and recurrence, as increased oxidative metabolism is linked to increased tumor cell survival and proliferation (76). Through the inhibition of both energy production routes, cancer cell viability, hence, was significantly reduced.

## Conclusion

**Figure 11:**
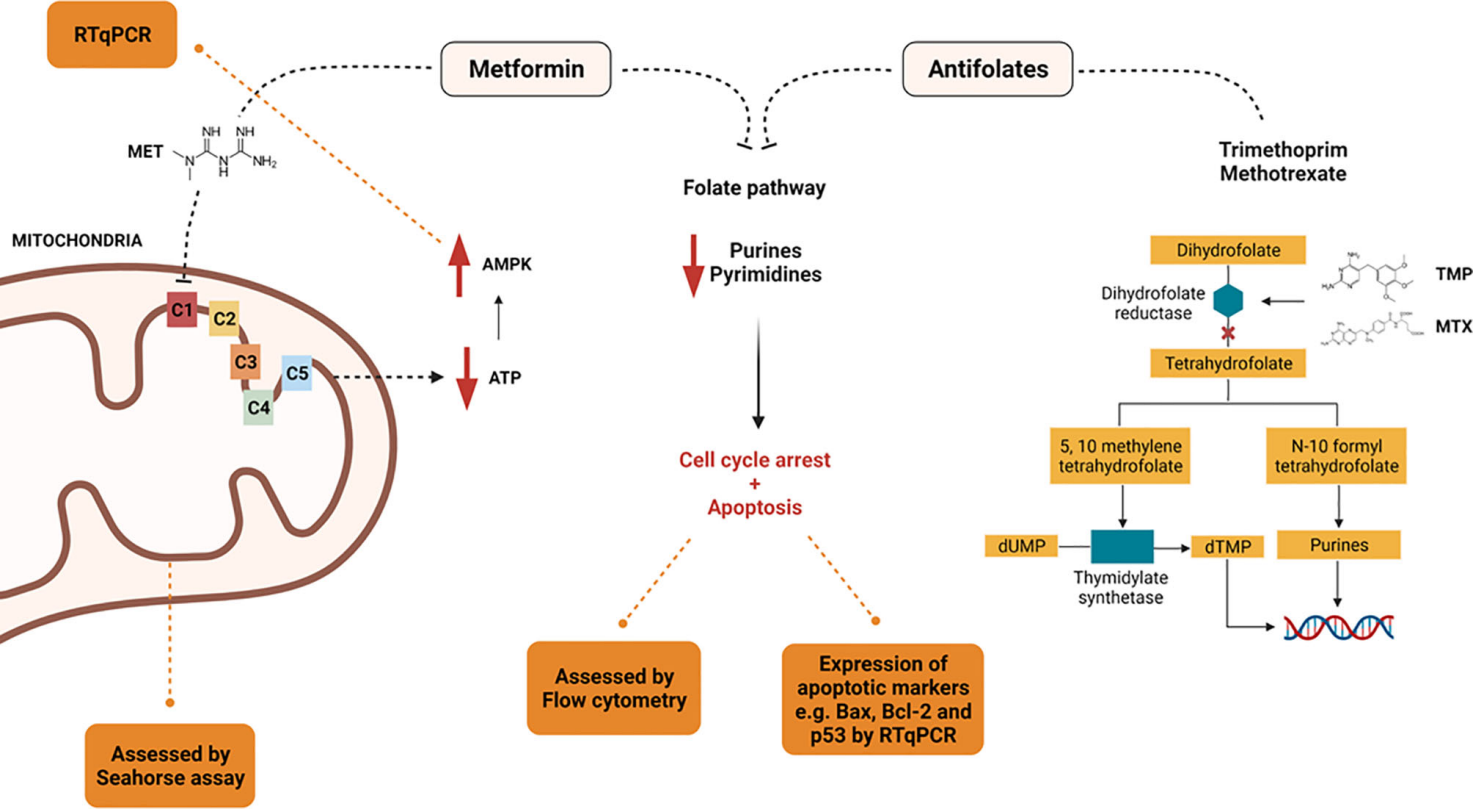
Summary of the proposed mechanisms by which MET, TMP, and MTX exert their effect.

To the best of our knowledge, no prior studies have been performed examining the bioenergic effects of combining MET with either TMP or MTX. In this study, the effects of MET alone as opposed to both combinations were compared, underlying the mechanisms involved in this combination *in vitro* on HepG2 cell line (**Figure 11**). Our data suggest that treatment of HepG2 cells with a combination of MET and antifolate agent (TMP or MTX) increases cell death than MET alone *via* mitochondrial inhibition and relative decrease in glycolysis. We suggest that the anti-cancer effect of MET combined with either antifolate agent occurs through the inhibition of cancer cell progression, increase expression of p53 and Bax, decrease expression of Bcl-2, rise in the number of total apoptotic cells, inhibition of migration ability, decrease in ATP production, inhibition of the glycolysis pathway and induction of mitochondrial damage.

## Data Availability Statement

The raw data supporting the conclusions of this article will be made available by the authors, without undue reservation.

## Author Contributions

Experiments and data collection were performed by ST, AA, and MA. Data analysis were carried out by ST, AA, MA, and ME. The study was designed by AA, MA, and HE-F. The first draft of the manuscript was written by ST and all authors revised the previous versions of the manuscript. The revised manuscript was written by ST and edited by AA and MA. All authors read and approved the final manuscript.

## Funding

This work was supported by AUC graduate research grant and AUC internal grant [FY19-RG (1-18)], Egyptian Academy of Scientific Research and Technology Grants (JESOR-2019-5305), and (ASRT-2019-4903), a Bartlett Fund for Critical Challenges Grant and an AUC COVID-19 Pandemic Research & Innovation Initiative Grant. Part of the work was funded by the British University in Egypt Young Investigator Research Grant (BUE-YIRG2018-07).

## Conflict of Interest

The authors declare that the research was conducted in the absence of any commercial or financial relationships that could be construed as a potential conflict of interest.

## Publisher’s Note

All claims expressed in this article are solely those of the authors and do not necessarily represent those of their affiliated organizations, or those of the publisher, the editors and the reviewers. Any product that may be evaluated in this article, or claim that may be made by its manufacturer, is not guaranteed or endorsed by the publisher.
